# Effects of tofacitinib monotherapy on patient-reported outcomes in a randomized phase 3 study of patients with active rheumatoid arthritis and inadequate responses to DMARDs

**DOI:** 10.1186/s13075-015-0825-9

**Published:** 2015-11-04

**Authors:** Vibeke Strand, Joel Kremer, Gene Wallenstein, Keith S. Kanik, Carol Connell, David Gruben, Samuel H. Zwillich, Roy Fleischmann

**Affiliations:** Biopharmaceutical Consultant, Portola Valley, CA USA; Center for Rheumatology, Albany Medical College, Albany, NY USA; Pfizer Inc, 445 Eastern Point Road, MS 8260-2515, Groton, CT 06340 USA; Metroplex Clinical Research Center, Dallas, TX USA; Stanford University, Palo Alto, CA USA

**Keywords:** Autoimmunity, Rheumatoid arthritis, Patient perspective

## Abstract

**Introduction:**

Tofacitinib is an oral Janus kinase inhibitor for the treatment of rheumatoid arthritis.

**Method:**

In this 6-month, phase 3, randomized, placebo-controlled trial, 611 patients with inadequate response to disease-modifying anti-rheumatic drugs (DMARD-IR) were randomized 4:4:1:1 to receive: tofacitinib 5 mg BID or tofacitinib 10 mg BID for the duration of the study, or placebo for 3 months followed by tofacitinib 5 mg BID or tofacitinib 10 mg BID. Patient-reported outcomes (PROs) included: Patient Global Assessment of Disease Activity (PtGA); Patient Assessment of Pain (Pain); Health Assessment Questionnaire-Disability Index (HAQ-DI); Medical Outcomes Survey (MOS) Short Form-36 (SF-36); Functional Assessment of Chronic Illness Therapy-Fatigue (FACIT-F); and MOS Sleep Scale. Time-to-event data (PtGA and Pain) were collected using an interactive voice response system daily diary (baseline through day 14).

**Results:**

At month 3, tofacitinib 5 and 10 mg BID demonstrated statistically significant improvements versus placebo in PtGA (both *p* < 0.0001), Pain (both *p* < 0.0001), HAQ-DI (both *p* < 0.0001), SF-36 Physical (*p* < 0.0001) and Mental (*p* < 0.05 [5 mg BID] and *p* < 0.0001 [10 mg BID]), Component Summary scores and all domain scores (*p* < 0.05–*p* < 0.0001) and FACIT-F (both *p* < 0.0001). Statistically significant changes from baseline in MOS Sleep Scale were reported for 10 mg BID (*p* < 0.05). Benefits of tofacitinib treatment were rapid in onset and significant improvements were reported at week 2 for PtGA, Pain and HAQ-DI, and differentiation from baseline was seen as early as 3 days after treatment initiation for interactive voice response system (IVRS) PtGA and IVRS Pain. The numbers needed to treat for patients to report changes greater than or equal to the minimum clinically important difference in PtGA, Pain, HAQ-DI, SF-36 Physical Component Summary score and FACIT-F ranged between 4.0–6.1 (5 mg BID) and 3.2–5.0 (10 mg BID).

**Conclusion:**

Tofacitinib monotherapy in DMARD-IR patients resulted in statistically significant and clinically meaningful improvements in multiple PROs versus placebo at month 3, with sustained improvements over 6 months.

**Trial registration:**

Clinicaltrials.gov registration NCT00814307, registered 22 December 2008

**Electronic supplementary material:**

The online version of this article (doi:10.1186/s13075-015-0825-9) contains supplementary material, which is available to authorized users.

## Introduction

Rheumatoid arthritis (RA) is a chronic autoimmune disease, which is characterized by systemic inflammation, persistent synovitis and joint destruction. RA represents a significant health and socioeconomic burden, and affects all domains of health-related quality of life (HRQoL), in particular physical functioning, pain, fatigue and physical and emotional roles [1, 2], which patients report are more important to them than joint counts and laboratory tests [3]. Both the US Food and Drug Administration and European Medicines Agency emphasize that patient-reported outcomes (PROs) selected for evaluation in a randomized controlled trial (RCT) should be targeted to the specific patient population [4, 5]. The Outcomes Measures in Rheumatology (OMERACT) international consensus effort recommends use of disease-specific and generic instruments to assess physical function, HRQoL and fatigue in RCTs in RA [3, 6–8].

The goal of therapy should be to use a treat-to-target strategy to achieve remission, if possible, or low disease activity, while limiting joint destruction, maintaining physical function and optimizing HRQoL [9]. Standard-of-care treatment includes conventional nonbiologic and/or biologic disease-modifying antirheumatic drugs (cDMARDs and bDMARDs, respectively) [10]. Patients who do not achieve remission, or low disease activity with advanced disease, with methotrexate and/or other cDMARDs are often escalated to treatment with bDMARDs (often in combination with cDMARDs), including cytokine inhibitors (tumor necrosis factor inhibitors, interleukin (IL)-6 inhibitors, IL-1 inhibitors), B cell inhibitors and B-T cell co-stimulation modulators [9, 11, 12]. As not all patients respond adequately to these medications, an unmet need for additional therapies persists, including those with alternative mechanisms of action.

Tofacitinib is an oral Janus kinase (JAK) inhibitor which preferentially inhibits signaling by heterodimeric receptors associated with JAK3 and/or JAK1, with functional selectivity over those that signal via pairs of JAK2 [13].

Administration of tofacitinib 5 and 10 mg orally twice daily (BID) has demonstrated sustained efficacy with a manageable safety profile in patients with RA in phase 2 [14–18] and phase 3 [19–24] RCTs of up to 24 months’ duration, and in long-term extension studies for up to 7 years [25, 26].

The phase 3 ORAL Solo RCT (A3921045) was designed to assess the efficacy and safety of tofacitinib monotherapy in patients with active RA, who had prior inadequate responses to cDMARDs or bDMARDs. The primary efficacy (including HAQ-DI) and safety data have been reported elsewhere [21], and showed that tofacitinib monotherapy results in reductions in signs and symptoms of active RA including improvement in physical function, with a manageable safety profile over 6 months. Here we present the complete profile of PROs from this phase 3 trial.

## Methods

### Study design and treatment

This was a phase 3, 6-month, placebo-controlled RCT (ClinicalTrials.gov NCT00814307; ORAL Solo), conducted at 94 centers worldwide (February 2009 to June 2010) in compliance with the Declaration of Helsinki and International Conference on Harmonisation Good Clinical Practice Guidelines. The final protocol was approved by Institutional Review Boards and/or Independent Ethics Committees at the investigational sites (Additional file 1: Table S1). Patients provided written, informed consent.

Details of the trial design and patient population are reported elsewhere [21]. Eligible patients were ≥18 years old, with RA for ≥6 months diagnosed by the American College of Rheumatology (ACR) 1987 Revised Criteria, with active disease (≥6 tender joints and ≥6 swollen joints; erythrocyte sedimentation rate >28 mm/h (measured in the local laboratory); and/or C-reactive protein >7 mg/L). Patients were required to have prior inadequate responses and/or intolerability to ≥1 cDMARD or bDMARD (499 patients (82.3 %) had prior inadequate response to methotrexate). A 4-week washout of failed DMARDs was required (12 weeks for abatacept and tocilizumab). Stable doses of antimalarial drugs, non-steroidal anti-inflammatory drugs (NSAIDs), and corticosteroids (≤10 mg/day prednisone equivalent) were permitted.

Patients were randomized 4:4:1:1 to receive tofacitinib 5 mg BID or tofacitinib 10 mg BID, or placebo for 3 months followed by tofacitinib 5 mg BID or tofacitinib 10 mg BID. At month 3, all placebo patients were switched blindly to active treatment and received tofacitinib for the next 3 months. Randomization was performed using an automated web/telephone system (Impala, Pfizer Inc, USA, New York, NY). The study was patient-blinded, investigator-blinded and sponsor-blinded.

### Assessment of patient-reported outcomes

Patient Global Assessment of Disease Activity (PtGA) and Patient Assessment of Pain (Pain) were evaluated using the 100-mm visual analog scale (VAS). Physical function was evaluated by the Health Assessment Questionnaire-Disability Index (HAQ-DI); HRQoL was evaluated by the Medical Outcomes Survey (MOS) Short Form-36 (SF-36; Version 2, Acute) questionnaire, which assesses eight domains (scores range from 0–100, with higher scores indicating better HRQoL): physical functioning (PF), role physical (RP), bodily pain (BP), general health (GH), vitality (VT), social functioning (SF), role emotional (RE), and mental health (MH). *Z*-transformed and normalized domain scores are grouped into Physical Component Summary (PCS) and Mental Component Summary (MCS) scores. The Functional Assessment of Chronic Illness Therapy-Fatigue (FACIT-F) scale was used to assess fatigue/tiredness and the MOS Sleep Scale, the quality of sleep.

PtGA, Pain, and HAQ-DI were assessed at all time points (baseline, week 2, months 1, 2, 3, 4, 5, and 6, and/or early termination). Time-to-event data were collected using an interactive voice response system (IVRS) daily diary from baseline through day 14 for PtGA (IVRS) and IVRS Pain. Time-to-event data were only collected from patients in the USA. The SF-36, FACIT-F, and MOS Sleep Scale were assessed at baseline, months 3 and 6, and/or early termination. Changes from baseline were compared with published values for minimum clinically important differences (MCID): ≥10 points in VAS PtGA and Pain [27–30], ≥0.22 points in HAQ-DI [27], ≥2.5 points, and ≥5 points in SF-36 summary and domain scores, respectively, [31–36] and ≥4 points in FACIT-F [37]. No MCID values have been determined for the MOS Sleep Scale.

### Disease activity

The Disease Activity Score based on C-reactive protein and 28 tender joint count and 28 swollen joint count (DAS28-3(CRP)) was used for comparison with results from PROs, because DAS28-3(CRP) is not composed of any of the PROs of interest.

### Statistical analyses

This manuscript focuses on the month 3 time point before placebo patients were switched to tofacitinib; analyses at 6 months examined whether improvements at 3 months in those receiving active therapy were sustained, and assessed changes following blinded switching of placebo patients to tofacitinib at 3 months. All analyses were based on the full analysis set, including all randomized patients who received at least one dose of study drug (modified intention to treat) with at least one post-baseline measurement. Furthermore, if a variable was expressed as change from baseline then there had to have been a non-missing baseline value.

Mean changes from baseline in continuous end points were expressed as least squares mean (LSM), and analyzed using a mixed-effects longitudinal model, which included effects of treatment and visit (geographic region of the investigative site, baseline value of the dependent variable, and the treatment-by-visit interaction), while patients were treated as a random effect.

The percentage of patients reporting improvements MCID was compared between tofacitinib and placebo groups using a normal approximation to the binomial (by forming a *z* score) to calculate numbers needed to treat (NNT). NNT was considered to be statistically significant if the percentage of responders by the MCID differed statistically from placebo. Pearson correlations of changes from baseline at month 3 in DAS28-3(CRP) with changes from baseline in HAQ-DI, Pain, and PtGA were calculated.

Statistical significance was declared for *p* ≤0.05, with no adjustment for multiple comparisons. Formal statistical comparisons between tofacitinib 5 and 10 mg BID dose groups were not performed as the study was not powered for these subgroup comparisons.

## Results

### Patients

Between February 2009 and June 2010, 611 patients were randomized to receive tofacitinib 5 mg BID (n = 244), tofacitinib 10 mg BID (n = 245), placebo followed by tofacitinib 5 mg BID (n = 61), and placebo followed by tofacitinib 10 mg BID (n = 61); 610 patients received at least one dose of study drug and 555 (91.0 %) completed the trial [21]. Most patients were Caucasian and female, with a mean age of 49.7–52.4 years across treatment groups and mean disease duration of 7.7–8.6 years [21].

**Fig. 1:**
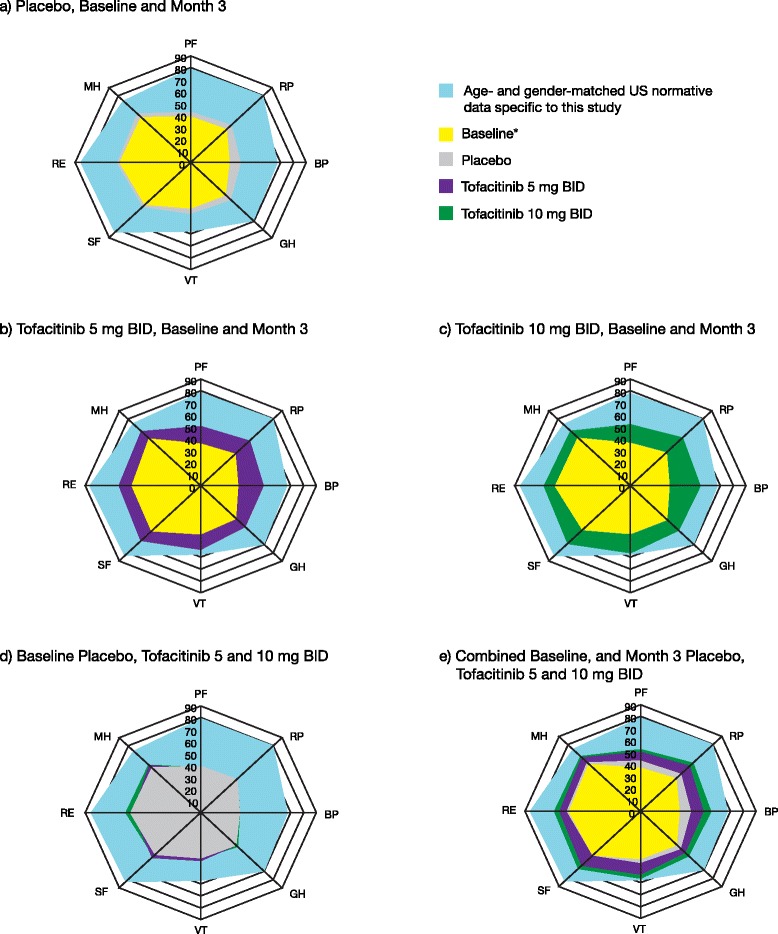
Short Form-36 (*SF-36*) domain scores at month 3. Spydergrams of SF-36 domain scores at month 3, with a US age- and gender-matched normative population as a comparator. **a** Placebo at baseline and month 3. **b** Tofacitinib 5 mg twice daily (*BID*) at baseline and month 3. **c** Tofacitinib 10 mg BID at baseline and month 3. **d** Baseline scores for each treatment group. **e** Weighted combined baseline score + placebo + tofacitinib 5 mg BID + tofacitinib 10 mg BID. **a**-**d** Treatment group baselines (using separate colors in **d**). **e** Weighted combined baseline across all treatment groups. Placebo, n = 122 at baseline, and n = 108 at month 3; tofacitinib 5 mg BID, n = 239 at baseline and n = 235 at month 3; tofacitinib 10 mg, n = 243 at baseline and n = 224 at month 3. Study values were normalized using means and SDs. See Ware et al. [38]. *BP* bodily pain, *GH* general health, *MH* mental health, *PF* physical function, *RE* role emotional, *RP* role physical, *SF* social functioning, *VT* vitality

Baseline values for SF-36 PCS and MCS scores were approximately 2 SD and 1 SD (10 points) below the normative value of 50 points (based on age- and gender-matched US normative data specific to this study population). Baseline domain scores were lowest in PF and RP domains (47–49 points lower than age- and gender-matched normative data), followed by BP and GH domains (34–36 points lower), RE, SF, MH and VT. Age- and gender-matched US normative data specific to this study population are plotted in Fig. 1 and baseline domain scores are presented in Table 1 [38, 39].

**Table 1:** Baseline values and changes from baseline at months 3 and 6 for patient-reported outcome measures

	Baseline mean (SD)			Month 3 LSM change from baseline (SE)			Month 6 LSM change from baseline (SE)			
	Placebo (n = 122)	Tofacitinib 5 mg BID (n = 243)	Tofacitinib 10 mg BID (n = 245)	Placebo (n = 122)	Tofacitinib 5 mg BID (n = 243)	Tofacitinib 10 mg BID (n = 245)	Placebo → Tofacitinib 5 mg BID (n = 61)	Placebo → Tofacitinib 10 mg BID (n = 61)	Tofacitinib 5 mg BID (n = 243)	Tofacitinib 10 mg BID (n = 245)
PtGA	62.63 (21.91)	61.66 (22.00)	63.46 (23.23)	−11.19 (2.10)	−26.99 (1.45)***	−30.94 (1.47)***	−28.07 (2.97)	−29.62 (3.05)	−30.25 (1.47)	−33.99 (1.50)
Pain	61.79 (21.27)	61.35 (22.27)	62.03 (23.63)	−10.71 (2.14)	−26.94 (1.47)***	−31.06 (1.50)***	−26.01 (3.02)	−29.48 (3.10)	−29.19 (1.49)	−34.11 (1.52)
HAQ-DI^†^	1.53 (0.65)	1.53 (0.66)	1.50 (0.64)	−0.19 (0.05)	−0.50 (0.03)***	−0.57 (0.03)***	−0.43 (0.07)	−0.59 (0.07)	−0.62 (0.03)	−0.67 (0.04)
SF-36 component summary scores										
SF-36 PCS score	32.21 (8.35)	31.23 (8.03)	31.37 (7.39)	2.63 (0.78)	6.79 (0.53)***	8.55 (0.55)***	5.18 (1.09)	6.14 (1.11)	8.01 (0.53)	9.66 (0.55)
SF-36 MCS score	39.87 (11.62)	41.36 (11.68)	42.19 (12.44)	1.09 (0.89)	4.11 (0.61)*	5.39 (0.62)***	3.83 (1.23)	8.01 (1.26)	4.62 (0.60)	4.80 (0.62)
SF-36 domain scores										
Physical functioning	31.41 (9.66)	30.09 (9.31)	30.51 (8.76)	2.17 (0.88)	6.15 (0.60)**	6.97 (0.62)***	4.02 (1.22)	5.15 (1.26)	6.95 (0.60)	8.17 (0.62)
Role physical	33.33 (8.97)	32.84 (8.87)	33.45 (8.69)	1.88 (0.83)	5.89 (0.57)***	7.53 (0.58)***	4.33 (1.16)	6.31 (1.20)	7.01 (0.57)	7.85 (0.59)
Bodily pain	32.77 (7.67)	32.41 (7.57)	32.74 (7.55)	3.91 (0.86)	8.26 (0.58)***	10.84 (0.60)***	7.88 (1.21)	10.28 (1.25)	9.44 (0.59)	11.30 (0.61)
General health	34.70 (9.00)	34.86 (8.72)	35.71 (8.86)	2.44 (0.75)	4.76 (0.51)*	6.34 (0.53)***	5.26 (1.06)	5.37 (1.10)	6.51 (0.52)	7.49 (0.54)
Vitality	40.13 (9.82)	41.22 (10.06)	41.04 (10.27)	2.03 (0.81)	6.56 (0.55)***	8.49 (0.57)***	4.67 (1.15)	8.27 (1.18)	6.87 (0.56)	8.30 (0.58)
Social functioning	35.16 (10.34)	36.78 (11.04)	36.07 (11.27)	0.62 (0.91)	5.29 (0.62)***	7.51 (0.64)***	3.54 (1.29)	8.21 (1.34)	6.05 (0.63)	6.83 (0.65)
Role emotional	35.17 (13.11)	34.26 (12.63)	36.70 (13.03)	1.20 (1.05)	4.07 (0.72)*	5.50 (0.74)**	4.54 (1.43)	6.96 (1.47)	5.95 (0.70)	5.56 (0.72)
Mental health	38.43 (12.36)	40.05 (11.49)	40.50 (12.57)	2.19 (0.88)	4.71 (0.60)*	5.52 (0.62)*	4.09 (1.23)	8.02 (1.27)	4.52 (0.60)	5.35 (0.62)
FACIT-F	27.17 (10.88)	27.90 (10.70)	27.72 (11.15)	2.84 (0.82)	6.70 (0.56)***	8.01 (0.58)***	6.57 (1.16)	9.11 (1.20)	6.98 (0.57)	8.63 (0.58)
MOS Sleep Scale	47.32 (21.24)	42.45 (18.38)	43.09 (20.41)	−4.81 (1.48)	−7.13 (1.02)	−10.18 (1.04)*	−8.43 (2.06)	−10.50 (2.14)	−7.48 (1.02)	−9.98 (1.05)

^†^Co-primary endpoint at month 3. *p < 0.05; **p < 0.001; *** *p* < 0.0001 vs placebo. *BID* twice daily, *FACIT-F* Functional Assessment of Chronic Illness Therapy-Fatigue, *HAQ-DI* Health Assessment Questionnaire-Disability Index, *LSM* least squares mean, *MCS* Mental Component Summary, *MOS* Medical Outcomes Study, *Pain* Patient Global Assessment of Pain, *PCS* Physical Component Summary, *PtGA* Patient Global Assessment of Disease Activity, *SD* standard deviation, *SE* standard error, *SF-36* Short Form-36

### Patient-reported outcomes

#### Patient global assessment of disease activity

**Fig. 2:**
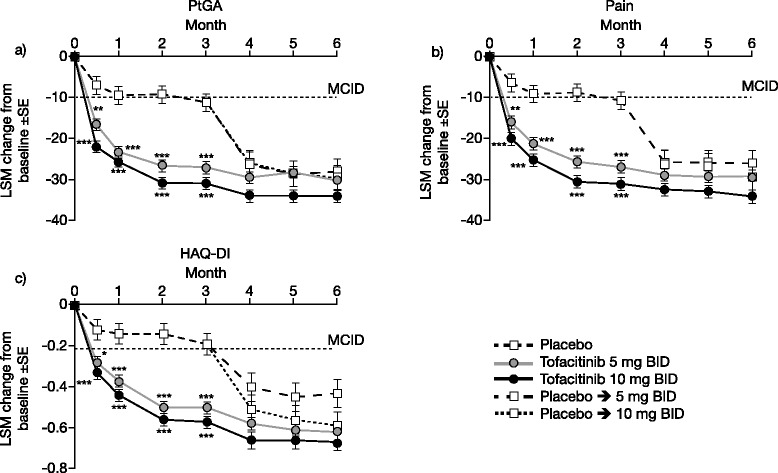
Least squares mean (*LSM*) changes from baseline: Patient Global Assessment of Disease Activity (*PtG*A) (**a**), Patient Global Assessment of Pain (*Pain*) (**b**), and Health Assessment Questionnaire-Disability Index (*HAQ-DI*) (**c**), over time; *p <0.05; **p <0.01; ***p <0.0001 vs placebo. MCID minimum clinically important difference, *BID* twice daily, *SE* standard error

**Fig. 3:**
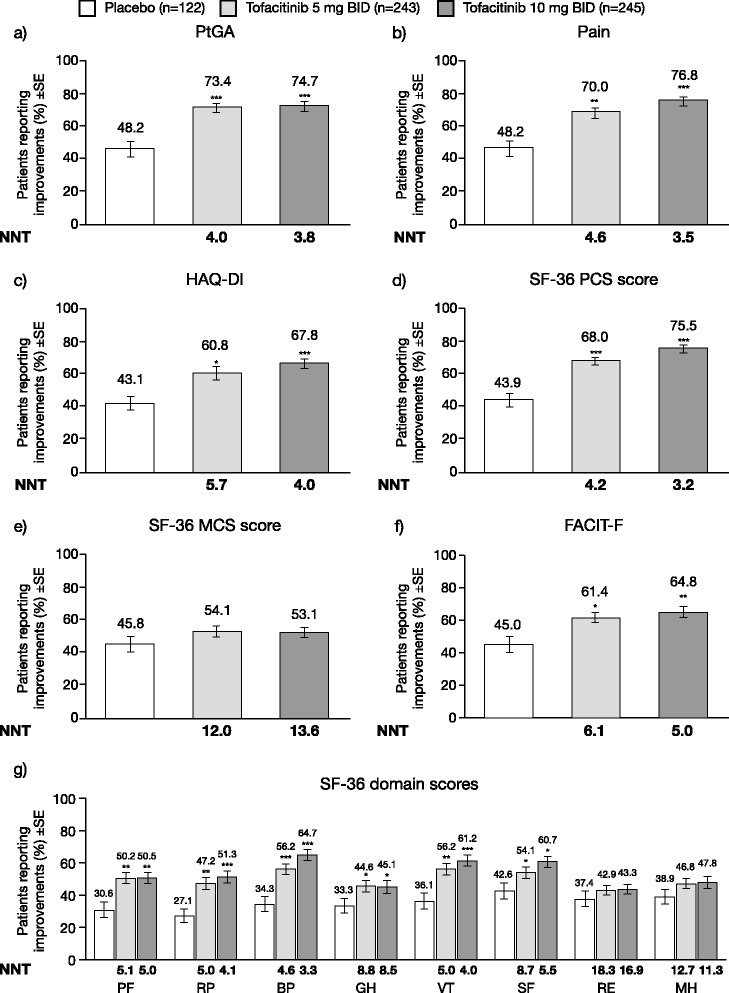
Percentage of patients with improvements ≥ the minimal clinically important difference (*MCID*) at month 3. Patient Global Assessment of Disease Activity (*PtGA*) (**a**), Patient Global Assessment of Pain (*Pain*) (**b**), Health Assessment Questionnaire-Disability Index (*HAQ-DI*) (**c**), Short Form-36 Physical Component Summary (*SF-36 PCS*) score (**d**), SF-36 Mental Component Summary (*MCS*) score (**e**), Functional Assessment of Chronic Illness Therapy-Fatigue (*FACIT-F*) (**f**) and SF-36 domain scores (**g**); *p <0.05; **p <0.01; ***p <0.0001 vs placebo. MCID ≥10 points in PtGA and Pain, ≥0.22 points in HAQ-DI, ≥2.5 points and ≥5 points in SF-36 summary and domain scores, respectively and ≥4 points in FACIT-F. *BID* twice daily, *BP* bodily pain, *GH* general health, *MH* mental health, *NNT* number needed to treat, *PF* physical function, *RE* role emotional, *RP* role physical, *SE* standard error, *SF* social functioning, *VT* vitality

**Fig. 4:**
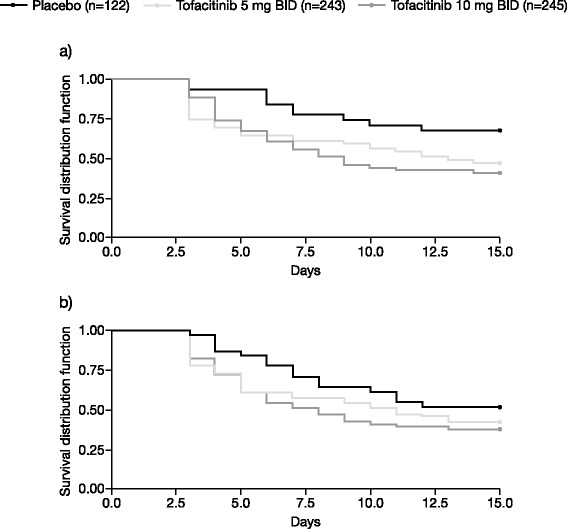
Time to first sequential decrease from baseline. Interactive voice response system (*IVRS*) Patient Global Assessment of Disease Activity (*PtGA*) (**a**) and IVRS Patient Global Assessment of Pain (*Pain*) (**b**). A sequential decrease was defined as at least 2 days of consecutive improvement in each measure. *BID* twice daily

At month 3, LSM changes from baseline were statistically significant with tofacitinib 5 and 10 mg BID treatment versus placebo (*p* < 0.0001), and exceeded the MCID (≥10 points) (Table 1; Fig. 2a). Statistically significant changes from baseline were evident at week 2 and months 1 and 2 for both doses of tofacitinib versus placebo (Fig. 2a; Additional file 2: Table S2) and further improvements occurred through month 6. Significantly more patients receiving tofacitinib reported improvements ≥ the MCID versus placebo (Fig 3a; Additional file 3: Table S3). Sequential decreases from baseline in IVRS PtGA occurred from approximately 3 days post-baseline (Fig. 4a), with greatest improvements observed in the 10 mg BID group from 6–15 days post-baseline. Placebo patients who were switched to tofacitinib reported clinically meaningful changes between months 3 and 6 (Table 1). Improvements and the percentage of patients reporting improvements ≥ the MCID at month 3 were numerically greater for 10 mg BID compared with 5 mg BID, with a lower NNT (3.8 vs 4.0) (Fig. 3a; Additional file 3: Table S3). NNT over time for PtGA compared with ACR20/50/70 and DAS28-3(CRP) are shown in Additional file 4: Figure S1. Correlation at month 3 with DAS28-3(CRP) (both expressed as LSM changes from baseline) ranged from 0.32 (tofacitinib 10 mg BID) to 0.51 (placebo); all were statistically significant.

#### Pain

At month 3, LSM changes from baseline were statistically significant in patients receiving tofacitinib 5 and 10 mg BID versus placebo (*p* < 0.0001), and exceeded the MCID (Table 1; Fig. 2b). Statistically significant changes from baseline were also reported at week 2 and months 1 and 2 for both doses of tofacitinib versus placebo (Fig. 2b; Additional file 2: Table S2) and further improvement occurred through month 6 in both active treatment groups (Table 1). Significantly more patients receiving tofacitinib reported improvements ≥ the MCID versus placebo (Fig. 3b; Additional file 3: Table S3). Patients reported sequential decreases in IVRS Pain from approximately 3 days post-baseline (Fig. 4b); more patients in the 10 mg BID group reported sustained improvements from 4–13 days post-baseline but formal statistical analyses between 5 mg BID and 10 mg BID were not performed. Placebo patients who were switched to tofacitinib reported clinically meaningful changes between months 3 and 6 (Table 1). Improvements in LSM values and the percentage of patients reporting improvements ≥ the MCID at month 3 were numerically greater in the 10 mg BID group compared with 5 mg BID group, with a lower NNT (3.5 vs 4.6) (Table 1; Fig. 3b; Additional file 3: Table S3). The NNT over time for pain compared with ACR20/50/70 and DAS28-3(CRP) are shown in Additional file 4: Figure S1. Correlations with DAS28-3(CRP) at month 3 (both expressed as LSM changes from baseline) ranged from 0.32 (tofacitinib 10 mg BID) to 0.46 (placebo); all were statistically significant.

#### Health assessment questionnaire-disability index

At month 3, LSM changes from baseline were statistically significant for tofacitinib 5 and 10 mg BID versus placebo (*p* < 0.0001), and exceeded the MCID (≥0.22 points) (Table 1; Fig. 2c). Statistically significant changes from baseline were also reported at week 2 (first post-baseline assessment) and months 1 and 2 for tofacitinib 5 and 10 mg BID versus placebo (Fig. 2c; Additional file 2: Table S2) and further improvements accrued through month 6 (Table 1). Significantly more patients treated with tofacitinib reported improvements ≥ the MCID compared with placebo (Fig. 3c; Additional file 3: Table S3). Placebo patients advanced to tofacitinib reported clinically meaningful changes between month 3 and month 6 (Table 1). Improvements in LSM values and the percentage of patients reporting improvements ≥ the MCID at month 3 were numerically greater in the 10 mg BID compared with the 5 mg BID group, with a lower NNT (4.0 vs 5.7) (Table 1; Fig. 3c; Additional file 3: Table S3). The NNT over time for HAQ-DI compared with ACR20/50/70 and DAS28-3(CRP) are shown in Additional file 4: Figure S1. Numerically more patients reported values consistent with normative scores in HAQ-DI (≤0.5) with tofacitinib 5 and 10 mg BID versus placebo (Additional file 5: Table S4). Correlations with DAS28-3(CRP) at month 3 ranged from 0.37 with tofacitinib 5 mg BID to 0.47 with placebo; all were statistically significant.

#### Health-related quality of life assessed by Short Form-36

LSM changes from baseline in SF-36 PCS and MCS scores were statistically significant for 5 mg BID (*p* < 0.0001 and *p* < 0.05, respectively) and 10 mg BID (both *p* < 0.0001) compared with placebo at month 3 (first post-baseline assessment), and exceeded the MCID (≥2.5 points) (Table 1). Significantly more patients in the tofacitinib treatment groups reported improvements ≥ the MCID in PCS scores versus placebo (Fig. 3d); the percentage of patients reporting improvements ≥ the MCID in MCS scores were not statistically significant for the tofacitinib treatment groups versus placebo (Fig. 3e). LSM changes from baseline and the percentage of patients reporting improvements ≥ the MCID at month 3 were numerically greater for 10 mg BID compared with 5 mg BID, with a lower NNT (PCS: 3.2 vs 4.2; MCS: 12.0 vs 13.6) (Table 1; Fig. 3d, e).

Changes from baseline in SF-36 domain scores compared with age and gender US normative data are presented in Fig. 1. At month 3, patients receiving tofacitinib reported statistically significant (*p* < 0.05 to *p* < 0.0001) and clinically meaningful improvements (≥5 points) from baseline in all domain scores (Table 1). Compared with placebo, significantly more patients receiving tofacitinib reported improvements ≥ the MCID (≥5 points) in the PF, RP, BP GH, VT, and SF domains (Fig. 3g). The percentage of patients reporting improvements meeting or exceeding US normative SF-36 scores for both tofacitinib doses compared with placebo are presented in Additional file 6: Figure S2.

#### Functional assessment of chronic illness therapy-fatigue

Statistically significant improvements from baseline were observed in the tofacitinib groups for FACIT-F (*p* < 0.0001) at month 3 (first post-baseline assessment) (Table 1). Significantly more patients receiving tofacitinib reported improvements ≥ the MCID (≥4 points) versus placebo (Fig. 3f). Further improvements occurred through month 6 in the active treatment groups (Table 1). Placebo patients who switched to tofacitinib reported clinically meaningful changes between months 3 and 6 (Table 1). LSM values and the percentage of patients reporting improvements ≥ the MCID at month 3 were numerically greater in the 10 mg BID group compared with the 5 mg BID group, with a lower NNT (5.0 vs 6.1) (Table 1; Fig. 3f).

#### Medical outcomes study sleep scale

Statistically significant changes from baseline in the MOS Sleep Scale were evident at month 3 (first post-baseline assessment) for tofacitinib 10 mg BID (*p* < 0.05) but not for tofacitinib 5 mg BID (p = 0.1926) versus placebo (Table 1). Further improvements occurred through month 6 in patients receiving active treatment, and similar changes from months 3–6 were reported by placebo patients who switched to tofacitinib (Table 1).

## Discussion

It has been demonstrated that PROs provide quantitative data of comparable value to more traditional measures (e.g., joint counts and laboratory tests), discriminate treatment effects, are easy to perform, and are important for long-term health outcomes [40]. Furthermore, HRQoL measures are unique in that they measure the impact of the underlying disease, treatment-related benefits and adverse effects, and offer the opportunity for comparison to other disease populations. PROs, clinical assessments and imaging of joints are all important in assessing RA patients and their responses to treatment, and should be utilized together to provide a holistic view of disease activity and wellbeing.

In this phase 3 trial, DMARD-IR patients receiving tofacitinib 5 and 10 mg BID monotherapy reported statistically significant and clinically meaningful improvements in PtGA, Pain, HAQ-DI, HRQoL, and fatigue at 3 months, with significant changes versus placebo observed at the first time point measured post-baseline, as early as 2 weeks. This is particularly important as patients were expected to have active disease at the time they initiated protocol treatment. In those patients continuing tofacitinib therapy, further improvements in LSM values were reported at month 6, and improvements in LSM values were greater for 10 versus 5 mg BID. Placebo patients who switched to tofacitinib at month 3 reported improvements through month 6, confirming the results of the primary analysis at 3 months. Benefits of treatment with tofacitinib, as demonstrated by these PROs, were consistent with primary efficacy data, which showed statistically significant improvements in ACR responses, and changes from baseline in HAQ-DI with tofacitinib 5 and 10 mg BID monotherapy versus placebo [21].

Improvements in PtGA, Pain, and HAQ-DI with tofacitinib versus placebo were similar, and consistent with changes reported for fatigue and HRQoL. Across all five of these PROs – PtGA, Pain, HAQ-DI, SF-36 PCS, and FACIT-F – the proportion of patients reporting improvements ≥ the MCID ranged from 61–73 % for 5 mg BID and 65–77 % for 10 mg BID. Improvements in LSM changes from baseline to months 3 and 6, and from 3 to 6 months in placebo patients switched to tofacitinib, were consistently greater for 10 mg BID versus 5 mg BID. Across these five PROs, the NNT values for treatment with 10 mg BID ranged from 3.2–5.0 compared with 4.0–6.1 for 5 mg BID, with such low numbers reflecting the value of therapy to patients.

In active RA, physical functioning, pain and fatigue have been shown to be important outcomes from the patient’s perspective [41] Tofacitinib therapy resulted in improvements in each of these aspects of the disease, measured by PtGA, Pain, HAQ-DI, FACIT-F, and the MOS Sleep Scale. Benefits of tofacitinib treatment were rapid in onset and significant improvements were reported at week 2 for PtGA, Pain, and HAQ-DI, and differentiation from baseline was seen as early as 3 days after treatment initiation for IVRS PtGA and IVRS Pain.

As expected, patients reported substantially diminished HRQoL at baseline, measured by SF-36, versus age- and gender-matched US normative data as a benchmark comparison (Fig. 1). This was particularly evident in the PF, RP, BP, GH, SF, and RE domains, consistent with the broad impact of active RA on physical, social, emotional, and mental functioning. Following tofacitinib treatment, patients reported statistically significant and clinically meaningful improvements in both summary scores and all domains of the SF-36 with both tofacitinib doses.

Statistically significant and clinically meaningful improvements in the VT domain resulted in scores that approached (5 mg BID) and met (10 mg BID) normative values from the US general population at month 3. These are reflected by improvements in the FACIT-F scores, which were statistically significantly greater versus placebo, and exceeded the MCID. Not only were changes from baseline greatest in those domains with the lowest scores at baseline (PF, RP, and BP), but improvements were also evident in other domains, including RE and MH, which correlate strongly with the classic instruments used to diagnose clinical depression. RA is known to have a major negative psychological impact, with depression occurring in 13–20 % of patients [42] or more [43] based on clinical assessments. Thus, it appears that tofacitinib not only improves physical functioning, pain and fatigue, but also social and emotional functioning and wellbeing.

Patients receiving placebo monotherapy improved at 3 months, although mean improvements were small in magnitude: below the MCID in the HAQ-DI and FACIT-F, and meeting the MCID in the PtGA, Pain, and PCS scores. Fewer than 50 % of placebo patients reported changes the MCID (43–48 %) in all PRO endpoints compared with ≥61 % and ≥68 % in the 5 and 10 mg BID groups, respectively.

## Conclusions

This phase 3 randomized clinical trial, ORAL Solo, demonstrates that treatment with tofacitinib monotherapy for 3 months provides relief from the broad burden of active RA, favorably impacting a wide range of PROs. These include self-assessment of physical function, pain, disease activity, and HRQoL, with low NNT and early onset of improvement in patients with a prior inadequate response to cDMARDs and/or bDMARDs.

## Additional files

Additional file 1: Table S1. Ethical review boards and study centers. (DOCX 35 kb) Additional file 2: Table S2. Baseline and changes from baseline by visit prior to month 3 for patient-reported outcome measures; **p* < 0.01; ***p* < 0.001; ****p* < 0.0001 vs placebo. ^a^n = 237; ^b^n = 240; ^c^n = 237; ^d^n = 238; ^e^n = 231; ^f^n = 241; ^g^n = 243. *BID* twice daily, *HAQ-DI* Health Assessment Questionnaire-Disability Index, *LSM* least squares mean, *Pain* Patient Global Assessment of Pain, *PtGA* Patient Global Assessment of Disease Activity, *SD* standard deviation, *SE* standard error. (DOCX 13 kb) Additional file 3: Table S3. Rates reports of improvements meeting or exceeding clinically meaningful and normative values in both a patient- reported outcome (PRO) and DAS28-(CRP) at month 3; ^*^ *p* ≤0.05, ^**^ *p* ≤0.01, ^***^ *p* ≤0.0001 versus placebo. *BI*D twice daily, *DAS28-3(CRP)* Disease Activity Score based on C reactive protein and 28 tender joint count and 28 swollen joint count, *HAQ-DI* Health Assessment Questionnaire-Disability Index, *NNT* number needed to treat versus placebo, *MCID* minimally clinically important difference. (DOCX 15 kb) Additional file 4: Figure S1. Numbers needed to treat for ACR 20/50/70 (**a**), Disease Activity Score based on C reactive protein and 28 tender joint count and 28 swollen joint count (*DAS28-3(CRP)*) improvement of 1.2 and 0.6 (**b**), HAQ-DI ≤0.5 (**c**), patient global assessment (**d**), and pain (**e**) ≥ the minimal clinically important difference (*MCID*) for tofacitinib 5 and 10 mg twice daily (*BID*). (DOC 146 kb) Additional file 5: Table S4. Rates of reporting normative values for the Health Assessment Questionnaire-Disability Index (*HAQ-DI*) (≤0.5) per visit; ^*^ *p* ≤0.05, ^**^ *p* ≤0.01, ^***^ *p* ≤0.0001 versus placebo. *BID* twice daily, *CI* confidence interval on the estimate of the rate. (DOCX 12 kb) Additional file 6: Figure S2. Rates of reporting improvements meeting or exceeding US normative Short Form-36 (*SF-36*) scores, in comparison with placebo. (DOC 120 kb)

## Abbreviations

ACR: American College of Rheumatology
bDMARD: biologic disease-modifying antirheumatic drug
BP: bodily pain
cDMARD: non-biologic DMARD
BID: twice daily
FACIT-F: Functional Assessment of Chronic Illness Therapy-Fatigue
GH: General health
HAQ-DI: Health Assessment Questionnaire-Disability Index
HRQoL: health-related quality of life
IVRS: interactive voice response system
LSM: least squares mean
MCID: minimum clinically important difference
MCS: Mental Component Summary
MH: mental health
MOS: Medical Outcomes Study
NNT: number needed to treat
NSAID: non-steroidal anti-inflammatory drug
Pain: Patient Global Assessment of Pain
PCS: Physical Component Summary
PF: physical functioning
PRO: patient-reported outcome
PtGA: Patient Global Assessment of Disease Activity
RA: rheumatoid arthritis
RE: role emotional
RP: role physical
SD: standard deviation
SE: standard error
SF: social functioning
SF-36: Short Form-36
VAS: visual analog scale
VT: vitality
