# Supplementary material for: Effects of tofacitinib monotherapy on patient-reported outcomes in a randomized phase 3 study of patients with active rheumatoid arthritis and inadequate responses to DMARDs
Source: Arthritis Res Ther. 2015 Nov 4;17:307. doi: 10.1186/s13075-015-0825-9 (PMC4632359; doi:10.1186/s13075-015-0825-9)
Supplement: Additional file 1: Table S1. — Ethical review boards and study centers. (DOCX 35 kb) [file 13075_2015_825_MOESM1_ESM.docx]

# Effects of tofacitinib monotherapy on patient-reported outcomes in a randomized Phase 3 study of patients with active rheumatoid arthritis and inadequate responses to DMARDs

Vibeke Strand, Joel Kremer, Gene Wallenstein, Keith S Kanik, Carol Connell, David Gruben, Samuel H Zwillich, Roy Fleischmann

**Additional file 1: Table S1.** Ethical review boards and study centers

| **Study Center** | **Institutional Review Board or Ethics Committee** |
| --- | --- |
| CEPIC - Centro Paulista de  Investigacao Clinica e Servicos  Medicos Ltda  Rua Moreira e Costa 342  Sao Paulo, SP 04266-010  BRAZIL | Hospital Heliopolis  Comite de Etica em Pesquisa  Rua Conego Xavier, 276. 10. andar  Sacoma  Sao Paulo, SP 04231-030  BRAZIL |
| CIP - Centro Internacional de  Pesquisas  Rua 9 B, 129 - 3 andar  Setor Oeste  Goiania, GO 74110-120  BRAZIL | Comite de Etica em Pesquisa do  Hospital Geral de Goiania -  CEPHGG  Avenida Anhhaguera, 6479 - Setor  Oeste  Goiania, GO 74110-010  BRAZIL |
| Hospital de Clinicas de Porto  Alegre  Servico de Reumatologia  Rua Ramiro Barcelos, 2350- 6º  andar - sala 645 A  Bom Fim  Porto Alegre, RS 90035-903  BRAZIL | Comite de Etica em Pesquisa em  Seres Humanos do Hospital de  Clinicas de Porto Alegre- HCPA  Rua Ramiro Barcelos 2350, 2 andar  Bom Fim  Porto Alegre, RS 90035-903  BRAZIL |
| Hospital de Clinicas de Porto  Alegre  Grupo de Pesquisa  Rua Ramiro Barcelos 2350 2  andar  sala 2202  Porto Alegre, RS 90035-003  BRAZIL |  |
| Centro de Estudos em Terapias  Inovadoras  Rua Padre Camargo, 241  Alto da Gloria  Curitiba, PR 80060-240  BRAZIL | Comitê de Ética em Pesquisa em  Seres Humanos do Hospital de  Clínicas - UFPR  Rua General Carneiro, 181  Curitiba, PR 80060-900  BRAZIL |
| CCBR Brasil  Centro de Pesquisas e Analises  Clinicas Ltda.  Rua Mena Barreto, 33  Rio de Janeiro, RJ 22271-100  BRAZIL | Comite de Etica em Pesquisa do  Hospital Pro Cardiaco Pronto Socorro  Cardiologico/PROCEP  Rua Dona Mariana, 219  Rio de Janeiro, RJ 22280-000  BRAZIL |
| Hospital Sao Lucas da PUCRS  Av. Ipiranga, 6690 - 4o andar  Porto Alegre, RS 90610-000  BRAZIL | Comite de Etica em Pesquisa da  Pontificia Universidade Catolica do  Rio Grande do Sul  Av. ipiranga 6690-Conj.314 -3 andar  Jardim Botanico  Porto Alegre, RS 90610-000  BRAZIL |
| IMA Brasil - Instituto de  Medicina Avancada  Praca Americo Jacomino, 55  Vila Madalena  Sao Paulo, SP 05437-010  BRAZIL | Comite de Etica em Pesquisa do  Instituto de Infectologia Emilio Ribas  Av. Dr. Arnaldo, 165  Sao Paulo, SP 01246-900  BRAZIL |
| MBAL na Voennomeditsinska  Akademia - Sofia  Klinika po Revmatologia i  Kardiologia  MMA HAT Sofia  Ul. "Georgi Sofiyski" 3  Sofia, 1606  BULGARIA | Ethic Committee for Multicenter  Trials  Etichna komisiya za mnogocentrovi  izpitvaniya  ul. "Damyan Gruev" 8  Sofia, 1303  BULGARIA |
|  | Komisiya po etika pri MBAL na  Voennomeditsinska Akademia -  Sofia/Ethics Commettee at MMA  HAT-Sofia  MMA HAT-Sofia  Ul. Georgi Sofiyski  3  Sofia, 1606  BULGARIA |
| DKTs "Sveta Anna" Sofia  Konsultativen Kabinet po  Revmatologia  Diagnostic Consultative Center  "Sveta Anna" Sofia  Ul. "Dimitar Mollov" 1  Sofia, 1709  BULGARIA | Ethic Committee for Multicenter  Trials  Etichna komisiya za mnogocentrovi  izpitvaniya  ul. "Damyan Gruev" 8  Sofia, 1303  BULGARIA |
|  | Komisiya po etika pri DKTs"Sv.  Anna"/ Ethics Committee at DCC  "Sv. Anna"  DKTs "Sveta Anna"  Diagnostic Consultative Center  "Sveta Anna"  Ul. Dimitar Mollov 1  Sofia, 1709  BULGARIA |
| MBAL "Sveti Ivan Rilski" Sofia  Klinika po Revmatologia  MHAT "Sveti Ivan Rilski"  Ul. "Urvich" 13  Sofia, 1612  BULGARIA | Ethic Committee for Multicenter  Trials  Etichna komisiya za mnogocentrovi  izpitvaniya  ul. "Damyan Gruev" 8  Sofia, 1303  BULGARIA |
| UMBAL "D-r Georgi Stranski"  Pleven  Klinika po Kardiologia i  Revmatologia  MHAT "Dr. Georgi Stranski"  Pleven  Ul. "Georgi Kochev" 8A  Pleven, 5800  BULGARIA | Ethic Committee for Multicenter  Trials  Etichna komisiya za mnogocentrovi  izpitvaniya  ul. "Damyan Gruev" 8  Sofia, 1303  BULGARIA |
|  | Komisiya po etika pri UMBAL"D-r  Georgi Stranski"/ Ethics Committee  at MHAT "Dr. Georgi Stranski"  UMBAL"D-r Georgi Stranski"  Pleven  MHAT "Dr. Georgi Stranski" Pleven  Ul. Georgi Kochev  8A  Pleven, 5800  BULGARIA |
| MBAL "Kaspela" Plovdiv,  Otdelenie po revmatologia  MHAT "Kaspela" Plovdiv  ul. Sofiya  64  Plovdiv, 4002  BULGARIA | Ethic Committee for Multicenter  Trials  Etichna komisiya za mnogocentrovi  izpitvaniya  ul. "Damyan Gruev" 8  Sofia, 1303  BULGARIA |
|  | Komisiya po etika kam MBAL  "Kaspela"/ Ethics committee at  MHAT "Kaspela"  MBAL 'Kaspela' Plovdiv  MHAT 'Kaspela' Plovdiv  Ul. Sofiya 64  Plovdiv, 4002  BULGARIA |
| MBAL - Plovdiv  Revmatologichno Otdelenie  MHAT - Plovdiv  bul. "Bulgaria" 234  Plovdiv, 4000  BULGARIA | Ethic Committee for Multicenter  Trials  Etichna komisiya za mnogocentrovi  izpitvaniya  ul. "Damyan Gruev" 8  Sofia, 1303  BULGARIA |
|  | Komisiya po etika pri MBALPlovdiv/  Ethics Committee at MHATPlovdiv  MBAL-Plovdiv  MHAT-Plovdiv  bul. Bulgaria 234  Plovdiv, 4000  BULGARIA |
| Consulta Privada Dra. Marta  Aliste  Guardia Vieja 255, Oficina 1409  Providencia  Santiago, RM 7510186  CHILE | Comite Etico Cientifico  Servicio de Salud Metropolitano  Oriente  Av. Salvador 364  Providencia, Santiago, RM 7500922  CHILE |
| Centro de Diagnostico y  Tratamiento San Borja Arriaran  Seccion Reumatologia  Amazonas 619  Santiago, RM 8360156  CHILE | Comite Etico Cientifico  Servicio de Salud Metropolitano  Oriente  Av. Salvador 364  Providencia, Santiago, RM 7500922  CHILE |
| Clínica Santa María, Sección  Reumatología  Fernando Manterola 0540  Providencia, Santiago, RM  7530206  CHILE | Comite de Etica  Clinica Santa Maria  Avenida Santa Maria 0410  Santiago,  CHILE |
|  | Comite Etico Cientifico  Servicio de Salud Metropolitano  Oriente  Av. Salvador 364  Providencia, Santiago, RM 7500922  CHILE |
| SERVIMED E.U  Calle 51# 34-17 Consultorio  208-208A Centro Comercial  Cabecera. Etapa I  Bucaramanga, Santander  COLOMBIA | Comite de ética en Investigación de  Servimed E.U  Calle 51 No. 34-17. Centro comercial  Cabecera Etapa I.  Bucaramanga, Santander  COLOMBIA |
| Centro de Reumatologia y  Ortopedia  Cra 49 C No 82-125  Barranquilla,  COLOMBIA | Comité de etica independiente centro  de reumatologia y ortopedia  Cr. 49C No. 82-120  Barranquilla, Atlantico 0000  COLOMBIA |
| Revmatologicka ambulance  Petra Rezka 3  Praha 4, 140 00  CZECH REPUBLIC | Eticka komise IKEM a FTNsP  Videnska 800  Praha 4 Krc, 140 59  CZECH REPUBLIC |
| Revmatologicka ambulance  Nuselska poliklinika  Taborska 57  Praha 4, 140 00  CZECH REPUBLIC | Eticka komise IKEM a FTNsP  Videnska 800  Praha 4 Krc, 140 59  CZECH REPUBLIC |
| L.K.N. Arthrocentrum, s.r.o.  Revmatologicka ambulance  Na Valech 1  Hlucin, 748 01  CZECH REPUBLIC | Eticka komise pri Fak. Thomayerove  nemocnici a IKEM  Videnska 800  Praha 4, 140 59  CZECH REPUBLIC |
| PV-Medical s.r.o.  Revmatologicka ambulance  Padelky I/3645  Zlin, 760 01  CZECH REPUBLIC | Eticka komise IKEM a FTNsP  Videnska 800  Praha 4 Krc, 140 59  CZECH REPUBLIC |
| ARTHROMED, s. r. o.  Revmatologicka ambulance  Rokycanova 2798  Pardubice, 530 02  CZECH REPUBLIC | Eticka komise IKEM a FTNsP  Videnska 800  Praha 4 Krc, 140 59  CZECH REPUBLIC |
| Revmatologicky ustav  Na Slupi 4  Praha 2, 128 50  CZECH REPUBLIC | Eticka komise  Revmatologicky ustav  Na Slupi 4  Praha 2, 128 50  CZECH REPUBLIC |
|  | Eticka komise IKEM a FTNsP  Videnska 800  Praha 4 Krc, 140 59  CZECH REPUBLIC |
| FN Brno, Interni  hematoonkologická klinika  Revmatologicka ambulance  Jihlavska 20  Brno, 625 00  CZECH REPUBLIC | Eticka komise FN Brno  Jihlavská 20  Brno, 625 00  CZECH REPUBLIC |
|  | Eticka komise IKEM a FTNsP  Videnska 800  Praha 4 Krc, 140 59  CZECH REPUBLIC |
| Revmatologie  Pod Holym vrchem 349  Ceska Lipa, 470 01  CZECH REPUBLIC | Eticka komise IKEM a FTNsP  Videnska 800  Praha 4 Krc, 140 59  CZECH REPUBLIC |
| Fakultni Thomayerova  nemocnice s poliklinikou  Revmatologicke a rehabilitacni  oddeleni  Videnska 800  Praha 4, 140 59  CZECH REPUBLIC | Eticka komise IKEM a FTNsP  Videnska 800  Praha 4 Krc, 140 59  CZECH REPUBLIC |
| Latin American Research  Avenida Maximo Gomez #60,  suite 201, plaza paseo del treatro  Santo Domingo, Santo Domingo  00000  DOMINICAN REPUBLIC | CONABIOS  Universidad Católica Santo Domingo  Calle Santo Domingo No3, Ens. La  Julia  Santo Domingo, Santo Domingo  2733  DOMINICAN REPUBLIC |
| Universitaetsklinik Leipzig,  Klinik fuer Gastroenterologie und  Rheumatologie, Sektion  Rheumatologie  Liebigstr. 20  Leipzig, 04103  GERMANY | Ethik-Kommission an der  Medizinischen Fakultaet der  Universitaet Leipzig  Haertelstrasse 16-18  Leipzig, 04107  GERMANY |
| Schlosspark-Klinik, Innere  Medizin II, Rheumatologie  Heubnerweg 2  Berlin, 14059  GERMANY |  |
| Schoen Klinik Hamburg - Eilbek,  Abt. Rheumatologie und Klin.  Immunologie  Dehnhaide 120  Hamburg, 22081  GERMANY |  |
| Klinikum der Universitaet  Muenchen, Campus Innenstadt  Pettenkoferstr. 8a  Muenchen, 80336  GERMANY |  |
| Arztpraxis, Internist -  Rheumatologie  Kontumazgarten 4  Nuernberg, 90429  GERMANY |  |
| FAE Innere Medizin /  Rheumatologie  Ludwig-Wucherer-Str. 10  Halle, 06108  GERMANY |  |
| St. John's Medical College  Hospital  Sarjapur Road  Bangalore, Karnataka 560 034  INDIA | Institutional Ethical Review Board  St. John's Medical College and  Hospital  Sarjapur Road  Bangalore, Karnataka 560 034  INDIA |
| Bowring and Lady Curzon  Hospitals  Shivaji Nagar  Bangalore, Karnataka 560 001  INDIA | Ethical Committee, Bangalore  Medical College & Research Institute  K.R. Road,  Fort,  Bangalore, Karnataka 560 002  INDIA |
| Chanre Rheumatology &  Immunology Centre & Research  #149, 15th Main NHCL  Water Tank Road  4th Block, 3rd Stage,  Basaveswaranagar  Bangalore, Karnataka 560 079  INDIA | Institutional Ethical Committee  Chanre Rheumatology &  Immunology Centre & Research  #149, 15th Main NHCL, Water Tank  Road, 4th Block, 3rd Stage  Basaveswaranagar  Bangalore, Karnataka 560 079  INDIA |
| Jehangir Clinical Development  Centre Pvt. Ltd.  Jehangir Hospital  32, Sassoon Road  Pune, Maharashtra 411 001  INDIA | Hirabai Cowasji Jehangir Medical  Research Institute and Jehangir  Clinical Development Center  Ethics Committee  Jehangir Hospital,  32, Sassoon Road,  Pune, Maharashtra 411 001  INDIA |
| Krishna Institute of Medical  Sciences Ltd  1-8-31/1  Minister Road  Secunderabad, Andra Pradesh  500 003  INDIA | Institutional Ethics Committee  Krishna Institute of Medical Sciences  Ltd.  1-8-31/1, Minister Road  Secunderabad, Andra Pradesh  500003  INDIA |
| Arthritis Research and Care  Foundation  Centre for Rheumatic Diseases  No. 11, Hermes Elegance  1988, Convent Street Camp  Pune, Maharashtra 411 001  INDIA | CRD Ethics Committee  11, Hermes Elegance  1988, Convent Street Camp  Pune, Maharashtra 411 001  INDIA |
| Shalby Hospitals  Opp. Karnavati Club  S.G. Road  P.O. Ambawadi Vistar  Ahmedabad, Gujarat 380 015  INDIA | Ethics Committee Shalby Hospitals  Shalby Hospitals  Opp. Karnavati Club,  S. G. Road, P. O. Ambawadi Vistar,  Ahmedabad, Gujarat 380 015  INDIA |
| Kasturba Medical College  Hospital  Attavar  Mangalore, Karnataka 575 001  INDIA | Manipal University Ethics Cimmittee  Madhav Nagar  Manipal, karnataka 576 104  INDIA |
| Father Muller Medical College  Fr. Muller Road,  Kankanady  Mangalore, Karnataka 575002  INDIA | Institutional Ethics Committee  Father Muller Medical College  Father Muller Road  Kankanady  Mangalore, Karnataka 575 002  INDIA |
| Sri Deepti Rheumatology Centre  6-2-45/8,  A.C.Guards,  Hyderabad, Andhra Pradesh 500  004  INDIA | Ethics Committee  Sri Deepti Rheumatology Centre  6-2-45/8,  A. C. Guards,  Hyderabad, Andhra Pradesh 500 004  INDIA |
| Mahavir Hospital & Research  Center  10-1-1, Bhagwan Mahavir Marg  A.C. Guards  Hyderabad, Andhra Pradesh 500  004  INDIA | Institutional Ethical Committee for  Bio Medical Research  Bhagwan Mahavir Medical Research  Center, Mahavir Hospital &  Research Center,  10-1-1, Bhagwan Mahavir Marg,  A.C. Guards,  Hyderabad, Andhra Pradesh 500 004  INDIA |
| Sarawak General Hospital  Jalan Hospital  Kuching, Sarawak 93586  MALAYSIA | Medical Research & Ethics  Committee  Ministry of Health, c/o NIH  Secretariat, Institute for Health  Management  Jalan Rumah Sakit  Bangsar  Kuala Lumpur, 59000  MALAYSIA |
| Queen Elizabeth Hospital  Kota Kinabalu, Sabah 88586  MALAYSIA |  |
| Hospital Putrajaya  Federal Government  Administration Centre  Presint 7  Putrajaya, Wilayah Persekutuan  62250  MALAYSIA |  |
| Sunway Medical Centre  No. 5, Jalan Lagoon Selatan  Bandar Sunway  Petaling Jaya, Selangor Darul  Ehsan 46150  MALAYSIA |  |
| Hospital Christus Muguerza del  Parque  Calle 14 1610 A Colonia Centro  Chihuahua, Chihuahua 31000  MEXICO | Comite de Etica e Investigacion del  Hospital Christus Muguerza del  Parque  Calle Dr. Pedro Leal Rodriguez y de  la Llave  Chihuahua, Chihuahua 31000  MEXICO |
| Hospital Universitario Jose  Eleuterio Gonzalez  Gonzalitos 235 Norte  Colonia Mitras Centro  Monterrey, Nuevo Leon 64020  MEXICO | Comite de Etica Facultad de  Medicina de la UANL y Hospital  Universitario Dr. Jose Eleuterio  Gonzalez  Av. Francisco I Madero Pte s/n y Dr.  E Aguirre Pequeno  Col. Mitras Centro  Monterrey, Nuevo Leon 64460  MEXICO |
| Unidad de Investigacion en  Enfermedades Cronico  Degenerativas  Colomos 2292  Colonia Providencia  Guadalajara, Jalisco 44620  MEXICO | Comite de Bioetica de la Unidad de  Investigacion en Enfermedades  Cronico-Degenerativas  Colomos 2292  Col. Providencia  Guadalajara, Jalisco 44620  MEXICO |
| Centro de Investigacion del  Noroeste SC  Boulevard Sanchez Taboada  9250 Interior 28  Zona Rio  Tijuana, Baja California 22010  MEXICO | Comision de Investigacion y Etica  Centro Medico Nova  Avenida Guadalupe Victoria 9308  Zona Rio  Tijuana, Baja California 22010  MEXICO |
| University of Santo Tomas  Hospital  6th Floor Hospital Research  Center  Clinical Division Building  Espana Street  Manila, Phlippines 1008  PHILIPPINES | Institutional Review Board  3rd Floor, Clinical Division Building  University of Sto. Tomas Hospital  Espana Blvd.  Manila, 1008  PHILIPPINES |
| University of Perpetual Help  Rizal Dalta Medical Center  7th Floor  Research Room  Alabang-Zapote Road, Pamplona  Las Piñas City, 1742  PHILIPPINES | Institutional Ethics Review Board  Institutional Ethics Review Board  University of Perpetual Help System-  DALTA 7th Floor Research Room  Alabang-Zapote Road  Las Piñas City, 1742  PHILIPPINES |
| De La Salle Health Sciences  Campus- Clinical Epidemiology  Unit  2nd Floor Clinical Trial Room#2  The Angelo King Medical  Research Center  Congressional Avenue  Dasmarinas  Cavite, Phlippines 4114  PHILIPPINES | DLS Institutional Review Board  Room 6301  De La Salle Angelo King Medical  Research Center  Congressional Avenue  Dasmariñas, Cavite 4114  PHILIPPINES |
| Southern Philippines Medical  Center  Section of Rheumatology  Department of Internal Medicine  Bajada, Davao City, Phlippines  8000  PHILIPPINES | Ethics Committee  Southern Philippines Medical Center  Bajada, Davao City, 8000  PHILIPPINES |
| Lecznica Specjalistow, Centrum  Medyczne "Osteomed" NZOZ  Al. Krakowska 110/114  Warszawa, 02-256  POLAND | Komisja Bioetyczna przy Okregowej  Izbie Lekarskiej w Warszawie  ul. Pulawska 18  Warszawa, 02-512  POLAND |
| "SYNEXUS SCM" Sp. z o.o.  ul. Swobodna 8a  Wroclaw, 50-088  POLAND |  |
| State Educational Institution of  Higher professional education  Smolensk State Medical  Academy  Roszdrav, Clinical Research  Centre of diagnostic medicine  and drugs  Krupskoy str., 28  Smolensk, 214019  RUSSIAN FEDERATION | Ethics Committee at the Federal  Service on Surveillance in Healthcare  and Social Development  8, str. 2, Petrovskij bulvar  Moscow, 127051  RUSSIAN FEDERATION |
|  | Independent Ethics Committee of  State Educational Institution of High  Professional Education  "Smolensk State Medical Academy  of Federal Agency of Healthcare and  Social Development"  28, ul. Krupskoj, 214019  27, pr. Gagarina  Smolensk, 214018  RUSSIAN FEDERATION |
| Republican Hospital n. a.  V.A.Baranov  Pirogova str., 3  Petrozavodsk, 185019  RUSSIAN FEDERATION | Ethics Committee at the Federal  Service on Surveillance in Healthcare  and Social Development  8, str. 2, Petrovskij bulvar  Moscow, 127051  RUSSIAN FEDERATION |
| City Clinical Hospital # 8, Dept  of Cardiology and Functional  Diagnostics  266g, Saltivske Shosse  Kharkiv, 61178  UKRAINE | Central Ethics Committee Ministry  of Health of Ukraine  5, Narodnogo Opolchennya Str.  Kyiv, 03680  UKRAINE |
|  | Committee for Ethics Issues of City  Clinical Hospital #8  266g, Saltivske Shosse  Kharkiv, 61178  UKRAINE |
| Vinnitsa Regional Clinical  Hospital n.a. Pirogov, Dept of  Internal Medicine #1 of Vinnitsa  NMU  46 Pirogova Street  Vinnitsa, 21018  UKRAINE | Bioethics Committee of Vinnitsa  Regional Clinical Hospital n.a.  Pirogov  46 Pirogova Street  Vinnitsa, 21018  UKRAINE |
|  | Central Ethics Committee Ministry  of Health of Ukraine  5, Narodnogo Opolchennya Str.  Kyiv, 03680  UKRAINE |
| Institute of Gerontology,  Department of Clinical  Physiology and Pathology of  Musculoskeletal System  67 Vyshgorodska Street  Kyiv, 04114  UKRAINE | Central Ethics Committee Ministry  of Health of Ukraine  5, Narodnogo Opolchennya Str.  Kyiv, 03680  UKRAINE |
|  | Committee for Ethics Issues of  Institute of Gerontology  67, Vyshgorodska Street  Kyiv, 04114  UKRAINE |
| Municipal City Clinical Hospital  #4  Department of Rheumatology  3 Sventsitskogo Street  Lviv, 79011  UKRAINE | Central Ethics Committee Ministry  of Health of Ukraine  5, Narodnogo Opolchennya Str.  Kyiv, 03680  UKRAINE |
|  | Committee for Ethics Issues of  Municipal City Clinical Hospital #4  3 Sventsitskogo Str  Lviv, 79011  UKRAINE |
| Republican Clinical Hospital  Dept of Internal Medicine #2 of  SI "Crimean State Medical  University n.a. S.I.  Georgiyevskyj"  69, Kyivska Street  Simferopol, Crimea, 95017  UKRAINE | Central Ethics Committee Ministry  of Health of Ukraine  5, Narodnogo Opolchennya Str.  Kyiv, 03680  UKRAINE |
|  | Committee for Ethics Issues of  Republican Clinical Hospital  69, Kyivska Street  Simferopol, Crimea 95017  UKRAINE |
| Clinical Research Center of  Reading, LLP  2760 Century Boulevard  Wyomissing, PA 19610  UNITED STATES | Quorum Institutional Review Board  Suite 1000  1601 Fifth Avenue  Seattle, WA 98101  UNITED STATES |
| Clinical Research Center of Cape  Cod, Inc.  131 Attucks Lane  Hyannis, MA 02601  UNITED STATES |  |
| East Penn Rheumatology  Associates, PC  Suite 501 & 601  701 Ostrum Street  Bethlehem, PA 18015  UNITED STATES |  |
| Florida Medical Clinic  Clinical Research Division  38135 Market Square  Zephyr Hills, FL 33542  UNITED STATES |  |
| Tampa Medical Group, PA  Suite 303  4700 North Habana Avenue  Tampa, FL 33614  UNITED STATES |  |
| STAT Research, Inc.  West Medical Plaza - Suite 230  One Elizabeth Place  Dayton, OH 45417  UNITED STATES |  |
| Arthritis Clinic & Carolina Bone  & Joint, PA  10460 Park Road  Charlotte, NC 28210  UNITED STATES |  |
| Mountain State Clinical Research  Suite 303A  300 Davisson Run Road  Clarksburg, WV 26301  UNITED STATES |  |
| United Hospital Center  X-ray and ECG Only  Suite 107  300 Davisson Run Road  Clarksburg, WV 26301  UNITED STATES |  |
| Altoona Center for Clinical  Research  175 Meadowbrook Lane  Duncansville, PA 16635  UNITED STATES |  |
| Investigational Drug Service  Drug Shipment Only  747 Broadway  Seattle, WA 98122  UNITED STATES |  |
| Seattle Rheumatology Associates  Suite 1000  1101 Madison  Seattle, WA 98104  UNITED STATES |  |
| Swedish Medical Center  747 Broadway  Seattle, WA 98122  UNITED STATES |  |
| Southwest Rheumatology, PA  Suite 615  18601 LBJ Freeway  Mesquite, TX 75150  UNITED STATES |  |
| Lovelace Scientific Resources  Suite C  411 Commercial Court  Venice, FL 34292  UNITED STATES |  |
| Venice Arthritis Center  Suite D  411 Commercial Court  Venice, FL 34292  UNITED STATES |  |
| Metroplex Clinical Research  Center  Suite 810  8144 Walnut Hill Lane  Dallas, TX 75231  UNITED STATES |  |
| AAIR Research Center  Suite 305  300 Meridian Centre  Rochester, NY 14618  UNITED STATES |  |
| The Center for Rheumatology,  LLP  Suite 101  1367 Washington Avenue  Albany, NY 12206  UNITED STATES |  |
| Jacksonville Center for Clinical  Research  Suite 1  4085 University Boulevard South  Jacksonville, FL 32216  UNITED STATES |  |
| Arthrocare, Arthritis Care and  Research PC  Suite 200  3921 East Baseline Road  Gilbert, AZ 85234  UNITED STATES |  |
| Rheumatology Associates of  North Jersey  1415 Queen Anne Road  Teaneck, NJ 07666  UNITED STATES |  |
| Boice-Willis Clinic, PA  Suite 320  901 North Winstead Avenue  Rocky Mount, NC 27804  UNITED STATES |  |
| Piedmont Arthritis Clinic, PA  Suite 400  3 St. Francis Drive  Greenville, SC 29601  UNITED STATES |  |
| Lovelace Scientific Resources  Suite 560  5741 Bee Ridge Road  Sarasota, FL 34233  UNITED STATES |  |
| The Arthritis Specialty Centre  Suite 550  5741 Bee Ridge Road  Sarasota, FL 34233  UNITED STATES |  |
| Rockford Orthopedic Associates,  Ltd.  324 Roxbury Road  Rockford, IL 61107  UNITED STATES |  |
| The Arthritis and Osteoporosis  Center of Maryland  71 Thomas Johnson Drive  Frederick, MD 21702  UNITED STATES |  |
| Carolina Health Specialists  Suite 4  945 82nd Parkway  Myrtle Beach, SC 29572  UNITED STATES |  |
| Catalina Pointe Clinical Research  Suite 100  7520 North Oracle Road  Tucson, AZ 85704  UNITED STATES |  |
| St. Joseph's Mercy Clinic  100 McGowan Court  Hot Springs, AR 71913  UNITED STATES | St. Joseph's Mercy Health Center  IRB  100 McAuley Court  Hot Springs, AR 71903  UNITED STATES |
| El Monte Mall - Suite 2010  652 Avenue Munoz Rivera  San Juan, PR 00918  UNITED STATES | Quorum Institutional Review Board  Suite 1000  1601 Fifth Avenue  Seattle, WA 98101  UNITED STATES |
| Baylor Research Institute  Arthritis Care and Research  Center  Suite 550  9900 North Central Expressway  Dallas, TX 75231  UNITED STATES | Baylor Research Institute  Institutional Review Board  White/Blue/Red  Suite 501  3310 Live Oak  Dallas, TX 75204  UNITED STATES |
| Trinity Health Center - Medical  Arts  400 Burdick Expressway East  Minot, ND 58701  UNITED STATES | Trinity IRB  Institutional Review Board  One Burdick Expressway West  Minot, ND 58701  UNITED STATES |
| Borgess Research Institute  Suites 003 and 004  1717 Shaffer Street  Kalamazoo, MI 49048  UNITED STATES | Quorum Institutional Review Board  Suite 1000  1601 Fifth Avenue  Seattle, WA 98101  UNITED STATES |
| Borgess Rheumatology  Suite 124  1717 Shaffer Street  Kalamazoo, MI 49048  UNITED STATES |  |
| Brookview Hills Internal  Medicine  Suite 207  3333 Brookview Hills Boulevard  Winston-Salem, NC 27103  UNITED STATES |  |
| Piedmont Medical Research  Associates  Suite 306  1901 South Hawthorne Road  Winston-Salem, NC 27103  UNITED STATES |  |
| Preferred Pain Management  Suite C  245 Charlois Boulevard  Winston-Salem, NC 27103  UNITED STATES |  |
| Southern Tier Arthritis and  Rheumatism  415 North 8th Street  Olean, NY 14760  UNITED STATES |  |
