# Supplementary material for: Effects of tofacitinib monotherapy on patient-reported outcomes in a randomized phase 3 study of patients with active rheumatoid arthritis and inadequate responses to DMARDs
Source: Arthritis Res Ther. 2015 Nov 4;17:307. doi: 10.1186/s13075-015-0825-9 (PMC4632359; doi:10.1186/s13075-015-0825-9)
Supplement: Additional file 2: Table S2. — Baseline and changes from baseline by visit prior to month 3 for patient-reported outcome measures; *p < 0.01; **p < 0.001; ***p < 0.0001 vs placebo. an = 237; bn = 240; cn = 237; dn = 238; en = 231; fn = 241; gn = 243. BID twice daily, HAQ-DI Health Assessment Questionnaire-Disability Index, LSM least squares mean, Pain Patient Global Assessment of Pain, PtGA Patient Global Assessment of Disease Activity, SD standard deviation, SE standard error. (DOCX 13 kb) [file 13075_2015_825_MOESM2_ESM.docx]

# Effects of tofacitinib monotherapy on patient-reported outcomes in a randomized Phase 3 study of patients with active rheumatoid arthritis and inadequate responses to DMARDs

Vibeke Strand, Joel Kremer, Gene Wallenstein, Keith S Kanik, Carol Connell, David Gruben, Samuel H Zwillich, Roy Fleischmann

**Additional file 2: Table S2.** Baseline and changes from baseline by visit prior to month 3 for patient-reported outcome measures

|  | **Baseline  mean (SD)** | | | **Week 2 LSM change from baseline (SE)** | | | **Month 1  LSM change from baseline (SE)** | | | **Month 2 LSM change from baseline (SE)** | | |
| --- | --- | --- | --- | --- | --- | --- | --- | --- | --- | --- | --- | --- |
|  | **Placebo (n=122)** | **Tofacitinib  5 mg  BID (n=243)** | **Tofacitinib 10 mg  BID (n=245)** | **Placebo (n= 119)** | **Tofacitinib  5 mg  BID (n=239)** | **Tofacitinib 10 mg  BID (n=239)** | **Placebo (n=116)** | **Tofacitinib  5 mg  BID (n=236)** | **Tofacitinib 10 mg  BID (n=240)** | **Placebo (n=110)** | **Tofacitinib 5 mg BID (n=239)** | **Tofacitinib 10 mg  BID (n=233)** |
| **PtGA** | 62.63 (21.91) | 61.66 (22.00)^b^ | 63.46 (23.23)^g^ | -7.02 (2.04) | -16.64 (1.44)^**^ | -22.01 (1.46)^***^ | -9.44 (2.06) | -23.26 (1.45)^***^ | -25.62 (1.45)^***^ | -9.22 (2.09) | -26.61 (1.44)^***^ | -30.81 (1.47)^***^ |
| **Pain** | 61.79 (21.27) | 61.35 (22.27)^f^ | 62.03 (23.63)^g^ | -6.32 (2.07) | -16.10 (1.47)^**^ | -20.03 (1.48)^***^ | -9.13 (2.09) | -21.28 (1.47)^***a^ | -25.25 (1.48)^***^ | -8.79 (2.13) | -25.65 (1.46)^***b^ | -30.58 (1.49)^***^ |
| **HAQ-DI** | 1.53 (0.65) | 1.53  (0.66)^b^ | 1.50  (0.64)^f^ | -0.12 (0.05) | -0.28 (0.03)^*^ | -0.33 (0.03)^**c^ | -0.14 (0.05) | -0.37 (0.03)^***^ | -0.44 (0.03)^***d^ | -0.14 (0.05) | -0.50 (0.03)^***^ | -0.56 (0.03)^***e^ |

*p<0.01; **p<0.001; ***p<0.0001 vs placebo.
^a^n=237, ^b^n=240, ^c^n=237, ^d^n=238, ^e^n=231; ^f^n=241; ^g^n=243

BID, twice daily; HAQ-DI, Health Assessment Questionnaire-Disability Index; LSM, least squares mean; Pain, Patient Global Assessment of Pain; PtGA, Patient Global Assessment of Disease Activity; SD, standard deviation; SE, standard error
