# Supplementary material for: Effects of tofacitinib monotherapy on patient-reported outcomes in a randomized phase 3 study of patients with active rheumatoid arthritis and inadequate responses to DMARDs
Source: Arthritis Res Ther. 2015 Nov 4;17:307. doi: 10.1186/s13075-015-0825-9 (PMC4632359; doi:10.1186/s13075-015-0825-9)
Supplement: Additional file 3: Table S3. — Rates reports of improvements meeting or exceeding clinically meaningful and normative values in both a patient- reported outcome (PRO) and DAS28-(CRP) at month 3; * p ≤0.05, ** p ≤0.01, *** p ≤0.0001 versus placebo. BID twice daily, DAS28-3(CRP) Disease Activity Score based on C reactive protein and 28 tender joint count and 28 swollen joint count, HAQ-DI Health Assessment Questionnaire-Disability Index, NNT number needed to treat versus placebo, MCID minimally clinically important difference. (DOCX 15 kb) [file 13075_2015_825_MOESM3_ESM.docx]

# Effects of tofacitinib monotherapy on patient-reported outcomes in a randomized Phase 3 study of patients with active rheumatoid arthritis and inadequate responses to DMARDs

Vibeke Strand, Joel Kremer, Gene Wallenstein, Keith S Kanik, Carol Connell, David Gruben, Samuel H Zwillich, Roy Fleischmann

**Additional file 3: Table 3.** Rates reports of improvements meeting or exceeding clinically meaningful and normative values in both a PRO and DAS28-(CRP) at month 3

|  |  |  | **Disease activity** | | | | | | | | |
| --- | --- | --- | --- | --- | --- | --- | --- | --- | --- | --- | --- |
|  |  |  | **Remission**  **DAS28-3(CRP) < 2.6** | | | **Low activity**  **DAS28-3(CRP) ≤ 3.2** | | | **Moderate activity**  **DAS28-3(CRP) ≤ 5.1** | | |
| **PRO** |  | **N** | **n** | **%** | **NNT** | **n** | **%** | **NNT** | **n** | **%** | **NNT** |
| **HAQ-DI ≤ 0.5** | **Tofacitinib 5 mg BID** | 238 | 23 | 9.7^*^ | 16.7 | 32 | 13.5^*^ | 12.6 | 63 | 26.5^*^ | 10.0 |
|  | **Tofacitinib 10 mg BID** | 229 | 33 | 14.4^**^ | 9.3 | 42 | 18.3^**^ | 7.8 | 63 | 27.5^*^ | 9.1 |
|  | **Placebo** | 109 | 4 | 3.7 | - | 6 | 5.5 | - | 18 | 16.5 | - |
| **Change from Baseline Pain ≥ MCID** | **Tofacitinib 5 mg BID** | 237 | 39 | 16.5^***^ | 7.8 | 52 | 21.9^***^ | 6.1 | 138 | 58.2^***^ | 4.2 |
|  | **Tofacitinib 10 mg BID** | 228 | 52 | 22.8^***^ | 5.2 | 76 | 33.3^***^ | 3.6 | 158 | 69.3^***^ | 2.9 |
|  | **Placebo** | 108 | 4 | 3.7 | - | 6 | 5.6 | - | 37 | 34.3 | - |
| **Change from Baseline Patient Global ≥ MCID** | **Tofacitinib 5 mg BID** | 237 | 35 | 14.8^**^ | 10.9 | 51 | 21.5^**^ | 7.1 | 145 | 61.2^***^ | 3.8 |
|  | **Tofacitinib 10 mg BID** | 229 | 49 | 21.4^***^ | 6.3 | 70 | 30.6^***^ | 4.3 | 153 | 66.8^***^ | 3.2 |
|  | **Placebo** | 108 | 6 | 5.6 |  | 8 | 7.4 |  | 38 | 35.2 |  |

^*^p≤0.05, ^**^p≤ 0.01, ^***^p<0.0001 versus placebo
BID, twice daily; DAS28-3(CRP); disease activity score based on C reactive protein and 28 tender joint count and 28 swollen joint count;
HAQ-DI, health assessment questionnaire – disability index; NNT number needed to treat, versus placebo; MCID minimally clinically effective difference; PRO, patient reported outcome
