# Supplementary figures and images for: Effects of tofacitinib monotherapy on patient-reported outcomes in a randomized phase 3 study of patients with active rheumatoid arthritis and inadequate responses to DMARDs
Source: Arthritis Res Ther. 2015 Nov 4;17:307. doi: 10.1186/s13075-015-0825-9 (PMC4632359; doi:10.1186/s13075-015-0825-9)

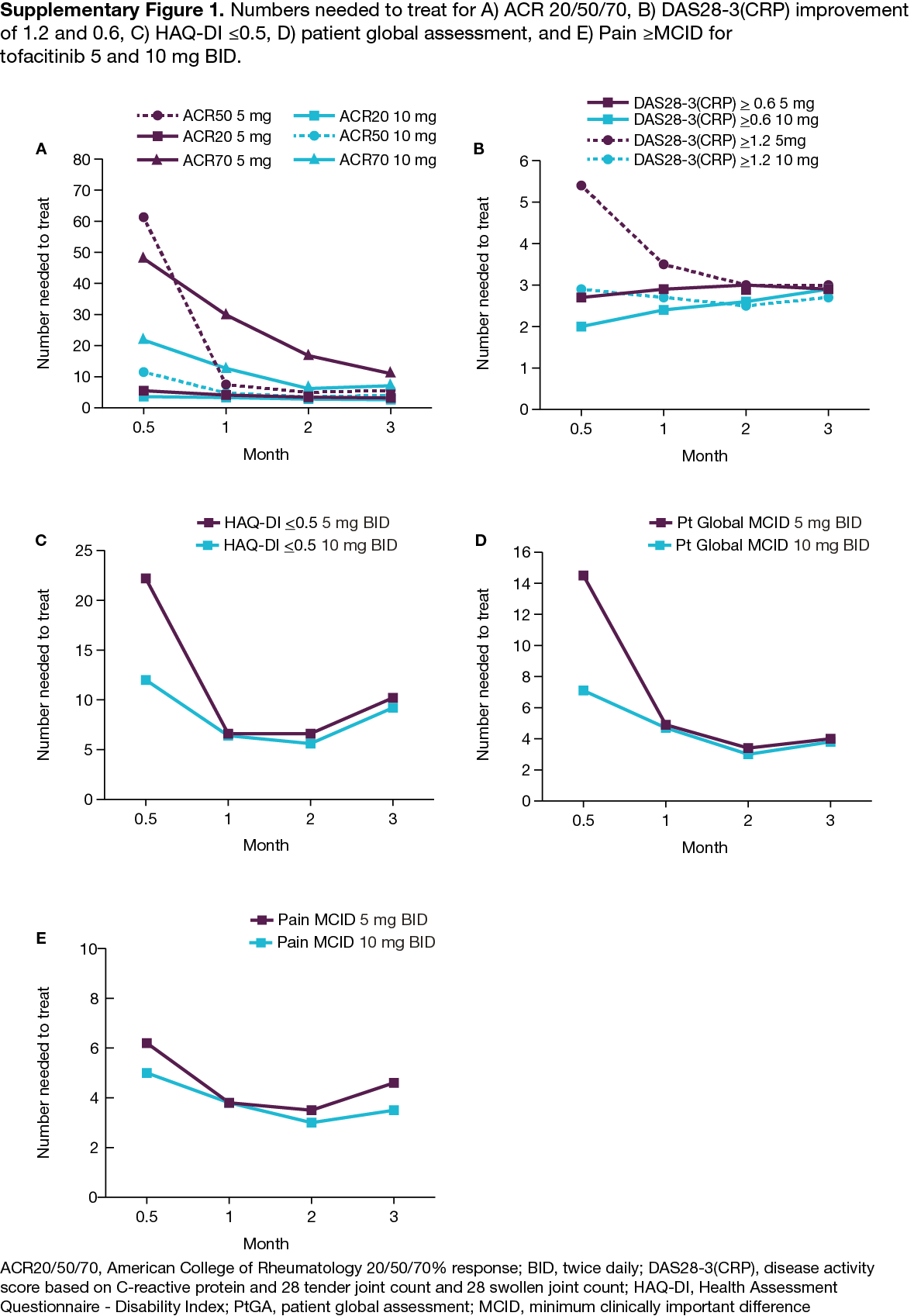

Supplement: Additional file 4: Figure S1. — Numbers needed to treat for ACR 20/50/70 (a), Disease Activity Score based on C reactive protein and 28 tender joint count and 28 swollen joint count (DAS28-3(CRP)) improvement of 1.2 and 0.6 (b), HAQ-DI ≤0.5 (c), patient global assessment (d), and pain (e) ≥ the minimal clinically important difference (MCID) for tofacitinib 5 and 10 mg twice daily (BID). (DOC 146 kb) [file 13075_2015_825_MOESM4_ESM.doc]

**
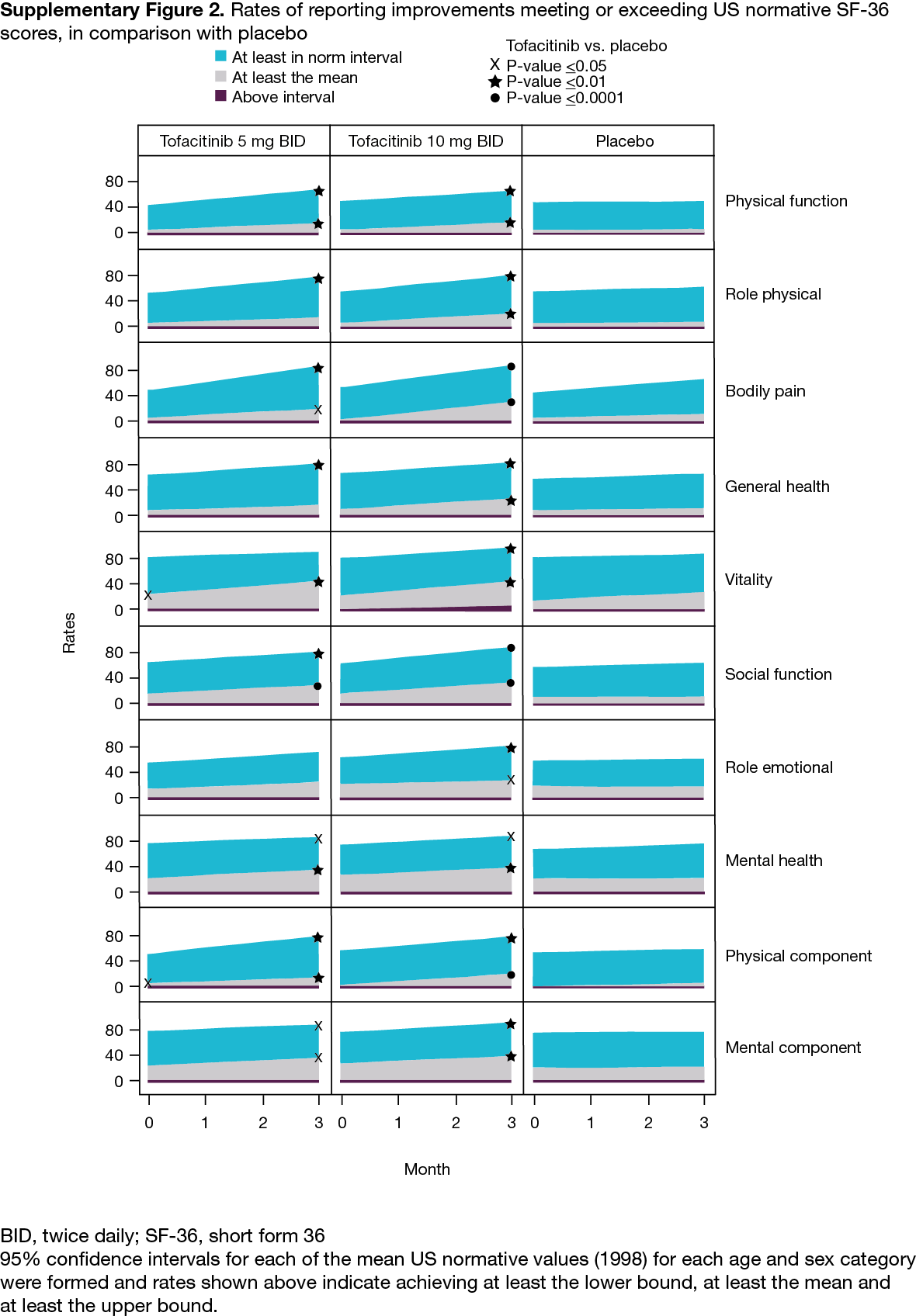
**

Supplement: Additional file 6: Figure S2. — Rates of reporting improvements meeting or exceeding US normative Short Form-36 (SF-36) scores, in comparison with placebo. (DOC 120 kb) [file 13075_2015_825_MOESM6_ESM.doc]
