# Supplementary material for: Effects of tofacitinib monotherapy on patient-reported outcomes in a randomized phase 3 study of patients with active rheumatoid arthritis and inadequate responses to DMARDs
Source: Arthritis Res Ther. 2015 Nov 4;17:307. doi: 10.1186/s13075-015-0825-9 (PMC4632359; doi:10.1186/s13075-015-0825-9)
Supplement: Additional file 5: Table S4. — Rates of reporting normative values for the Health Assessment Questionnaire-Disability Index (HAQ-DI) (≤0.5) per visit; * p ≤0.05, ** p ≤0.01, *** p ≤0.0001 versus placebo. BID twice daily, CI confidence interval on the estimate of the rate. (DOCX 12 kb) [file 13075_2015_825_MOESM5_ESM.docx]

# Effects of tofacitinib monotherapy on patient-reported outcomes in a randomized Phase 3 study of patients with active rheumatoid arthritis and inadequate responses to DMARDs

Vibeke Strand, Joel Kremer, Gene Wallenstein, Keith S Kanik, Carol Connell, David Gruben, Samuel H Zwillich, Roy Fleischmann

**Additional file 5: Table S4.** Rates of reporting normative values for HAQ-DI (≤0.5) per visit

|  |  | **N** | **n** | **Response rate (95% CI)** |
| --- | --- | --- | --- | --- |
| **0.5 months** | **Tofacitinib 5 mg BID** | 240 | 31 | 12.9 (8.7, 17.2) |
|  | **Tofacitinib 10 mg BID** | 239 | 40 | 16.7^*^ (12.0, 21.5) |
|  | **Placebo** | 119 | 10 | 8.4 (3.4, 13.4) |
| **1 month** | **Tofacitinib 5 mg BID** | 237 | 50 | 21.1^***^ (15.9, 26.3) |
|  | **Tofacitinib 10 mg BID** | 240 | 52 | 21.7^***^ (16.5, 26.9) |
|  | **Placebo** | 116 | 7 | 6.0 (1.7, 10.4) |
| **2 months** | **Tofacitinib 5 mg BID** | 240 | 65 | 27.1^**^ (21.5, 32.7) |
|  | **Tofacitinib 10 mg BID** | 233 | 69 | 29.6^***^ (23.8, 35.5) |
|  | **Placebo** | 110 | 13 | 11.8 (5.8, 17.9) |
| **3 months** | **Tofacitinib 5 mg BID** | 238 | 67 | 28.2^*^ (22.4, 33.9) |
|  | **Tofacitinib 10 mg BID** | 229 | 67 | 29.3^*^ (23.4, 35.2) |
|  | **Placebo** | 109 | 20 | 18.4 (11.1, 25.6) |

^*^p≤0.05, ^**^p≤0.01, ^***^p<0.0001 versus placebo

BID, twice daily; CI, confidence interval on the estimate of the rate, HAQ-DI, health assessment questionnaire – disability index
